# Associations between work-related stress in late midlife, educational attainment, and serious health problems in old age: a longitudinal study with over 20 years of follow-up

**DOI:** 10.1186/1471-2458-14-878

**Published:** 2014-08-27

**Authors:** Charlotta Nilsen, Ross Andel, Stefan Fors, Bettina Meinow, Alexander Darin Mattsson, Ingemar Kåreholt

**Affiliations:** Aging Research Center, Karolinska Institutet and Stockholm University, Stockholm, Sweden; School of Aging Studies, University of South Florida, Tampa, FL USA; International Clinical Research Center, St. Anne’s University Hospital, Brno, Czech Republic; Institute for Gerontology, School of Health Sciences, Jönköping University, Jönköping, Sweden

**Keywords:** Work-related stress, Psychosocial work environment, Socioeconomic position, Education, Multimorbidity, Complex health problems, Old age, Longitudinal, Sweden

## Abstract

**Background:**

People spend a considerable amount of time at work over the course of their lives, which makes the workplace important to health and aging. However, little is known about the potential long-term effects of work-related stress on late-life health. This study aims to examine work-related stress in late midlife and educational attainment in relation to serious health problems in old age.

**Methods:**

Data from nationally representative Swedish surveys were used in the analyses (n = 1,502). Follow-up time was 20–24 years. Logistic regressions were used to examine work-related stress (self-reported job demands, job control, and job strain) in relation to serious health problems measured as none, serious problems in one health domain, and serious problems in two or three health domains (complex health problems).

**Results:**

While not all results were statistically significant, high job demands were associated with higher odds of serious health problems among women but lower odds of serious health problems among men. Job control was negatively associated with serious health problems. The strongest association in this study was between high job strain and complex health problems. After adjustment for educational attainment some of the associations became statistically nonsignificant. However, high job demands, remained related to lower odds of serious problems in one health domain among men, and low job control remained associated with higher odds of complex health problems among men. High job demands were associated with lower odds of complex health problems among men with low education, but not among men with high education, or among women regardless of level of education.

**Conclusions:**

The results underscore the importance of work-related stress for long-term health. Modification to work environment to reduce work stress (e.g., providing opportunities for self-direction/monitoring levels of psychological job demands) may serve as a springboard for the development of preventive strategies to improve public health both before and after retirement.

## Background

People spend a considerable amount of time at work over the course of their lives, and this makes the workplace an important factor with respect to health and aging. However, little is known about the potential long-term effects of work-related stress on health problems in old age.

Overwhelming evidence indicates that unfavorable psychosocial working conditions are associated with poor health. The job demand-control model, constructed by Karasek [1], is an established model for measuring work-related psychological stress. The main premise of the model is that job strain arises from the combined effects of psychological job demands and control over the job situation [2]. Psychological demands refer to perceived workload and time pressure. Job control (also called decision latitude) is an interactive combination of decision-making authority and skill use. In particular, high job strain (high demands in combination with low control) is associated with an increased risk of stress and stress-related illnesses (e.g., coronary heart disease, musculoskeletal pain, and depressive symptoms [3–8]). Moreover, both low control and high demands are independently associated with coronary heart disease [5], psychological distress [9, 10], and poor self-rated health [9, 11, 12]. Conversely, high control may buffer the negative effects of high job demands [1]. Research shows that depression and musculoskeletal symptoms (e.g., aches and pain in the shoulders and neck) that are associated with psychological job demands and job strain are more prevalent in women than men. Men, however, are more mentally stressed by (e.g., experience anxiety because of) low control than women [6, 10, 13].

Furthermore, there is a social gradient in morbidity; i.e., a strong association between low socioeconomic position and poor health [14, 15], and research shows that favorable psychosocial working conditions mediate some of this association [11, 16]. But the relationship between socioeconomic position and work-related stress is far from straightforward. For example, lower socioeconomic position tends to be associated with poor psychosocial working conditions, such as low control [5, 17–19], whereas psychological job demands are often positively associated with higher socioeconomic position [3, 17, 20]. Finally, work-related stress has shown to not correlate that strongly with socioeconomic position [21]. Various indicators of socioeconomic position have been used in research. Education is a commonly used indicator of socioeconomic position and a major determinant of important socioeconomic conditions such as occupation and income. It aims to reflect intellectual and material resources derived from the possibilities that an education can give you in life [15]. For example, a person with high education may have her health less affected by a stressful work environment as she may possess more resources (material, cognitive, and psychosocial) that can be used to cope with the stressful conditions.

Most previous research on associations between work-related stress and health is cross-sectional, however, it may take years before physical consequences of work stress emerge, making it important that a long follow-up period be allowed to track the link between work stress and health. Few longitudinal studies have focused on the effects of such stress in old age (after retirement); however, associations between earlier work-related stress and health in old age have been observed in some studies. Work-related stress, measured as low control and high job strain, is associated with late-life health outcomes such as cognitive decline, dementia, and Alzheimer’s disease [22–24]. High psychological job demands are associated with worse physical health (e.g., physical function) and mental health (e.g., vitality and social functioning) after retirement [21, 25]. Low levels of control are associated with higher likelihood of multiple health problems in old age, including cognitive problems, physical limitations, and psychological distress [26]. Finally, psychological stress and work-related exhaustion may accelerate the biological aging process [27–29].

Although health problems are often simultaneous and interrelated in late life, especially among women [26, 30], most research has focused on single, specific conditions. Composite health measures based on a broad range of health problems — including diseases, symptoms and functional impairments — may provide a more nuanced picture of health problems in the older population.

The study aims to: (i) explore the associations between self-reported measures of work-related stress in midlife and serious health problems in old age, (ii) explore whether the associations between work-related stress and serious health problems in old age differ between the sexes, and (iii) explore whether the association between work-related stress and serious health problems in old age varies by level of education measured at baseline.

## Methods

### Data and study population

Data from two linked, nationally representative Swedish surveys were used in the analyses: the Level of Living Survey (LNU) and the Swedish Panel Study of Living Conditions of the Oldest Old (SWEOLD) [31, 32]. All six waves of LNU (1968, 1974, 1981, 1991, 2000, and 2010) were based on nationally representative samples of persons aged 18 through 75 years in Sweden. As they reach 76 years, persons interviewed in LNU are included in SWEOLD (SWEOLD has been conducted in four waves; 1992, 2002, 2004, and 2011. SWEOLD 2004 included persons 69+ years). Response rates in LNU range between 78 and 91 percent, and in SWEOLD, between 86 and 95 percent.

The information used in this study consisted of four linked sets of data combined into one longitudinal dataset. These linked sets of data included 1) baseline data from LNU 1968 plus re-interviews of the same people in SWEOLD 1992, 2) baseline data from LNU 1981 plus re-interviews of the same people in SWEOLD 2002, 3) baseline data from LNU 1981 plus re-interviews of the same people in SWEOLD 2004, and 4) baseline data from LNU 1991 plus re-interviews of the same people in 2011. Thus, the follow-up period ranged between 20 and 24 years. If respondents from baseline in 1968 or 1981 did not respond to any of the questions, the same respondent’s answer from LNU 1974 was used in the analyses. If respondents from baseline 1991 did not respond to any of the questions, the same respondent’s answer from LNU 1981 was used in the analyses. Such back-up information was rarely used and did not exceed 10 percent in any of the variables. Data were mainly collected through face-to-face interviews, with the exception of SWEOLD 2004 which was based on telephone interviews. Proxy interviews (e.g., a caregiver or family member) were conducted if direct interviews were not possible because of the individual’s health.

Students, housewives, long-term unemployed persons, and other persons without gainful occupation at baseline were excluded from the analyses because their working conditions could not be assessed. Persons who at baseline were over retirement age but were still working were excluded from the analyses. Age varied between 46 and 67 at baseline and 69 and 91 at follow-up. Retirement age in Sweden, which was 67 in 1968, was changed to 65 in 1976. The study included a total of 1,502 observations, 785 (52.3%) women and 717 (47.7%) men.

Written consent was obtained from all participants. A relative (normally a spouse or an adult offspring) signed the consent for participants who were physically too impaired at the time of the interview. The SWEOLD 1992 data collection was approved by Uppsala University Hospital Ethical Committee (Dnr 247/91), the SWEOLD 2002 data collection was approved by the Karolinska Institutet Ethical Research Committee (Dnr 03-413), the SWEOLD 2004 data collection was approved by the Regional Ethical Review Board in Stockholm (Dnr 04-314/5), and the SWEOLD 2011 data collection was also approved by the Regional Ethical Review Board in Stockholm (Dnr 2010/403-31/4).

### Measures of work-related stress

Two self-reported factors were used to measure work-related stress: job demands and job control. In accordance with Karasek’s job demand-control model, the measures were used both separately and in combination [1].

#### Job demands

Two questions about psychological job demands, which have been described and validated by Karasek and colleagues [2], were asked in the survey: “Is your work psychologically taxing/demanding?” and “Is your work hectic?” Participants who answered no to both questions were categorized as having low job demands. Those who answered yes to one question were categorized as having medium job demands and those who answered yes to both questions were categorized as having high job demands. Medium and high job demands were combined as high job demands, because analyses using a 3-level job demand variable (low-, medium-, and high job demands) showed that medium and high job demands resulted in approximately the same odds of health outcomes.

#### Job control

The job control variable was measured by self-reported evaluation of how repetitious/monotonous the participants’ work was and the skill level required (years of training normally required) for the job. We used four categories: 1) low control, defined as repetitious/monotonous work; 2) medium-low control, defined as work that was not repetitious/monotonous and that required a minimum skill level; 3) medium-high control, defined as work that was not repetitious/monotonous and that normally required 1 to 4 years of training; and 4) high control, defined as work that was not repetitious/monotonous and that normally required more than 4 years of training. A test using dummy variables for the job control categories confirmed that it was reasonable to include job control as a linear variable.

#### Job strain

Job strain was based on the cross-classification of job demands and job control. Job control was dichotomized by combining 1) low control and 2) medium-low control into low control and 3) medium-high control and 4) high control into high control, to measure job strain in four dimensions: low strain (high control/low demands), passive (low control/low demands), active (high control/high demands), and high strain (low control/high demands).

### Measures of serious health problems

In this study we focus on serious health problems in one or more of three important health domains: 1) diseases and symptoms during the last 12 months, 2) cognition and/or communication, and 3) mobility. The variable was coded as no serious problems in any domain, serious health problems in one domain, and complex health problems (designating serious problems in two or all three health domains). The concept of complex health problems follows work by Meinow and colleagues, and was originally constructed to capture the simultaneous presence of serious medical and functional problems indicating care needs from several different providers of both medical care and social services [33].

#### Diseases/symptoms

Participants were asked about thirteen diseases and/or symptoms, including general fatigue/sleeplessness, dizziness, leg ulcers, diabetes, stomachache, myocardial infarction/other heart problems, stroke, breathlessness, chest pain, hypertension, joint pain, back pain, and shoulder pain. Three responses were possible: “No”, “Yes, mild problems”, and “Yes, severe problems”. In our analyses, “No” was coded as 0, “Yes, mild problems” as 1, and “Yes, severe problems” as 3. BMI was assessed using self-reported weight and height and categorized as follows: <16 = severe underweight, coded as 3; between 16 and under 22 = mild underweight, coded as 1; ≥22 = not underweight, coded as 0. Underweight is considered to be a good predictor of mortality among old people [34]. A summed index that included diseases/symptoms and BMI was created that ranged from 0 to 42. A cut-off for serious problems for the diseases and symptom domain was determined at the highest quintile for the 1992 SWEOLD survey, corresponding to a score of 9 or higher on the summed scale. People scoring at this range had, for example, at least three severe symptoms/diseases or two severe and three mild. The same cut-off was used for the 2002, 2004, and 2011 SWEOLD samples.

#### Cognition/communication

An abridged version of the Mini-Mental State Examination (MMSE) [35] was used to measure cognition and ability to communicate. In the 1992, 2002, and 2011 follow-ups, the maximum score was 11 with a cut-off at 7. Participants who scored less than 7 were categorized as having cognitive impairment. This measure has been validated previously against clinical dementia diagnosis [36]. Since SWEOLD 2004 consisted only of telephone interviews one item that requires a face-to-face interview was excluded and thus the maximum was changed to 10 and the cut-off to 6. Interviewer notes showed that proxy-interviewed persons were too sick or weak to participate directly — the vast majority because of cognitive problems or, in a few cases, aphasia. Therefore, respondents who scored below the cut-off in the test, who did not take the test, or who could not be interviewed directly were coded as having serious cognitive and/or communication problems.

#### Mobility

The mobility domain in SWEOLD consisted of an index of four abilities: walking 100 meters fairly briskly without difficulties, walking up stairs, rising from a chair without using arms, and standing without support. Persons with limitations in at least three of the four activities were coded as having serious mobility problems.

### Measure of educational attainment

Education was measured at baseline by highest level of education.

#### Education

Education achieved was divided into four groups: compulsory, vocational, upper secondary, and university. First, we controlled for level of education given dummy representation including all four categories. Then, level of education was dichotomized as low education (compulsory; 6-8 years for most of the study participants) and high education (vocational, upper secondary, and university). Education was dichotomized due to the low number of persons with education above the compulsory level (compulsory 816 persons, vocational 557 persons, secondary 102 persons, and university 70 persons).

### Statistical analysis

Descriptive statistics were used to study the distribution and prevalence of work-related stress and level of education in the study population at baseline in relation to health problems at follow-up. Preliminary analyses showed that the variable number of health domains with serious health problems was a non-normally distributed, ordinal variable. Ordered logistic regression is suitable for ordinal variables like number of health domains with serious health problems if the assumption about proportional odds holds. Exploratory analyses with multinomial logistic regression showed that the assumption about proportional odds was violated, suggesting that using multinomial logistic regression was the better approach to analyze the available data. Therefore, multinomial logistic regressions were conducted to study the associations between work-related stress during midlife and number of health domains with serious health problems, analyzed as two separate outcomes in old age – serious problems in one health domain and serious problems in two or three health domains (what we call complex health problems). Binary logistic regression analyses were conducted to study the associations between work-related stress during midlife and the three different health domains separately.

In addition, we examined the interactions between a) work-related stress and level of education on the odds of having serious health problems in old age, in one or 2-3 health domains respectively, and b) work-related stress and sex on serious health problems in old age, in one and 2-3 health domains and each of the three different health domains separately. We used Spearman’s test for correlations between level education and work-related stress. All regression analyses were adjusted for follow-up year and baseline characteristics: age, sex, physical work environment (i.e., daily sweating, and exposure to vibrations, gas/smoke/dust, and poison/acid/explosive substances), hours worked during previous year, mental health (i.e., anxiety, depression, and general fatigue), mobility, and an index based on all diseases and symptoms that were used to create the outcome.

This study used baseline data from between 1968 and 1991 and combined four different data sets, in to one larger data set. Interaction effects between 1) follow-up year and work-related stress and 2) follow-up year and level of education were explored. This exploration showed that the associations between work-related stress or education and health problems were similar in all four linked data sets (1968 + 1992, 1981 + 2002, 1981 + 2004, and 1991 + 2011).

Observations about data on individuals who participated in both the 2002 and the 2004 surveys (n = 234) may be correlated because of the short period of time between the surveys. To avoid incorrect low standard errors, we used cluster-correlated robust estimate of variance, also known as Huber-White robust standard errors. This made it possible to use outcomes from 2002 and 2004 even if they were correlated [37].

## Results

Approximately 40 percent of the participants had serious problems in at least one health domain. Nearly 11 percent (12.7 percent of women and 8.5 percent of men) had serious problems in two or three health domains; i.e., complex health problems (Table 1).

**Table 1:** **Descriptive statistics**

	Number of domains with serious health problems				High education ^a^(%)
	None	1 domain	2-3 domains ^b^	Total	
**Sex** *(%)*					
Women	56.4	30.8	12.7	785	48.1
Men	67.4	24.1	8.5	717	55.4
**Age**					
Mean (baseline year)	55.6	56.9	58.6	56.3	55.9
Median	57.0	57.0	59.0	57.0	57.0
Mean (follow-up year)	80.6	81.1	81.7	80.9	80.4
Median	80.0	81.0	81.0	80.0	80.0
**Job demands** *(%)*					
Low demands	58.4	31.2	10.4	452	38.9
Medium demands	64.1	25.1	10.8	554	47.7
High demands	61.9	27.2	10.9	1496	67.8
**Job control** *(%)*					
Low control	52.1	33.1	14.8	263	30.2
Medium low control	59.3	28.3	12.4	605	29.5
Medium high control	63.6	27.0	9.4	415	74.9
High control	75.8	20.6	3.7	219	95.1
**High job strain** ^c^ *(%)*	57.3	29.1	13.6	546	34.0
**Level of education** *(%)*					
Low	54.9	31.6	13.5	725	
High	68.0	23.9	8.1	777 (51.7%)	51.7
TOTAL *n* ^d^ *(%)*	926 (61.7)	415 (27.6)	161 (10.7)	1502	

^a^Level of education was dichotomized as low education (compulsory; 6-8 years of education for most of the study participants) and high education (vocational, upper secondary, and university). ^b^Those with serious problems in two or three health domains were classified as having complex health problems. ^c^Job strain: Control was dichotomized into low and medium low vs. high and medium high. High strain = high demands and low control. ^d^Indicates total number of observations.

### Work-related stress and serious health problems

Among women, high job demands were associated with a higher likelihood of complex health problems (i.e., serious problems in at least two health domains) although this association was not statistically significant. Among men, however, the pattern was of high job demands and lower likelihood of serious health problems (in one or two or three domains) (Table 2, Model I). Low job control was associated with a higher likelihood of complex health problems among both women (p = .067) and among men (Table 2, Model I). Low control/high demands (high job strain) was associated with 2.62 times higher odds of complex health problems compared to low job strain (high control/low demands), when women and men were analyzed together (Table 2, Model I).

**Table 2:** **Associations between work-related stress and domains with serious health problems**

	Number of domains with serious health problems ***(ref. no serious problems)***			
	Model I		Model II	
WORK-RELATED STRESS	1 domain	2-3 domains^a^ (Complex health problems)	1 domain	2-3 domains, (Complex health problems)
**Job Demands** *(ref. low)* ^b^	OR (CI 95%)^c^	OR (CI 95%)	OR (CI 95%)	OR (CI 95%)
*Total*	0.76 (0.58-1.01)	1.00 (0.64-1.56)	0.82 (0.61-1.09)	1.10 (0.71-1.69)
*Women*	1.02 (0.69-1.51)	1.61 (0.88-2.97)	1.07 (0.71-1.61)	1.79 (0.98-3.27)
*Men*	**0.54** (0.36-0.81)	0.56 (0.29-1.08)	**0.59** (0.39-0.88)	0.60 (0.31-1.14)
Difference women/men^d^	*	*	*	*
**Job Control** *(linear* ^e^ *, high values = low control)*				
*Total*	1.15 (0.99-1.34)	**1.47** (1.18-1.84)	1.02 (0.86-1.22)	**1.30** (1.01-1.67)
*Women*	1.05 (0.85-1.28)	1.33 (0.98-1.80)	0.95 (0.75-1.20)	1.12 (0.81-1.54)
*Men*	**1.28** (1.01-1.62)	**1.68** (1.22-2.32)	1.13 (0.86-1.48)	**1.58** (1.08-2.30)
Difference women/men^d^	*ns*	*ns*	*ns*	*ns*
**High job strain** ^f^ *(ref. low strain* ^g^ *)*				
*Total*	0.79 (0.50-1.24)	**2.62** (1.03-6.69)	0.65 (0.41-1.04)	2.17 (0.82-5.74)
*Women*	0.70 (0.36-1.40)	3.12 (0.80-12.18)	0.59 (0.28-1.22)	2.14 (0.55-8.27)
*Men*	0.83 (0.44-1.57)	2.28 (0.60-8.66)	0.69 (0.36-1.32)	2.14 (0.51-8.96)
Difference women/men^d^	*ns*	*ns*	*ns*	*ns*

Results of multinomial logistic regressions. All analyses were adjusted for follow-up year and baseline characteristics: age, sex, physical work environment, hours worked during previous year, mental health, mobility, and an index based on all diseases and symptoms that were used to create the outcome. Model II was adjusted for all these factors and for level of education. The analyses were conducted separately for women and men. Results in bold: p value <0.05. ^a^Those with simultaneous problems in two or three health domains were classified as having complex health problems. ^b^For women, the reference category was women with low job demands. For men, the reference category was men with low job demands. ^c^Abbreviations: *OR* odds ratio, *CI* confidence interval, *ns* nonsignificant. ^d^P value for how the association between work-related stress and health problems differs between men and women; e.g., the interaction between sex and job demands: *p < 0.05, *ns* = *p* ≥ 0.05. ^e^In linear representation, the ORs indicate the change in odds of each higher category of the independent variable. Job control has four categories. ^f^High job strain: control was dichotomized into low and medium low vs. high and medium high. High strain = high demands and low control. ^g^For women, the reference category was women with low job strain. For men, the reference category was men with low job strain.

Job demands remained significantly associated with serious health problems in old age after additional adjustment for level of education: high job demands among men were associated with lower odds of serious problems in one health domain. Low job control among men remained significantly associated with higher odds of complex health problems. The association between job strain and complex health problems was reduced to nonsignificant after adjustment for level of education (Table 2, Model II). There were no interactions between sex and work-related stress that were associated with either serious problems in one health domain or with complex health problems, aside from job demands (Table 2, Model I and Model II).

When we analyzed the association between job demands and the serious problems in the three health domains one at a time, it became clear that there was an exception to the overall pattern (shown in Table 2) of a link between high job demands and higher odds of serious health problems in women but not in men. Demanding jobs seemed to protect both women and men from serious cognitive difficulties. Moreover, jobs with low control were associated with higher odds of serious health problems in all domains and in both sexes, but the association was only statistically significant with regard to mobility and cognition problems (Table 3, Model I).

**Table 3:** **The associations between work-related stress and serious health problems in old age by domain**

	Domain with serious health problems ***(ref. no serious problem in each of the domains respectively)***		
	Diseases/symptoms	Mobility	Cognition
WORK-RELATED STRESS	**Model I**		
**Job Demands** *(ref. low)* ^a^	OR (CI 95%)^b^	OR (CI 95%)	OR (CI 95%)
*Total*	1.00 (0.74-1.36)	1.10 (0.73-1.65)	**0.69** (0.50-0.95)
*Women*	1.37 (0.92-2.04)	1.54 (0.85-2.81)	0.77 (0.49-1.22)
*Men*	0.64 (0.40-1.01)	0.75 (0.42-1.34)	**0.56** (0.35-0.88)
Difference women/men^c^	*	*ns*	*ns*
**Job Control** *(linear * ^d^ *, high values = low control)*			
*Total*	1.10 (0.94-1.29)	**1.33** (1.09-1.64)	**1.36** (1.13-1.64)
*Women*	1.05 (0.86-1.28)	1.27 (0.95-1.70)	1.27 (0.98-1.64)
*Men*	1.20 (0.93-1.56)	**1.41** (1.06-1.89)	**1.47** (1.13-1.92)
Difference women/men^c^	*ns*	*ns*	*ns*
**High job strain** ^e^ *(ref. others)* ^f^			
*Total*	1.06 (0.79-1.42)	1.36 (0.92-2.00)	1.22 (0.88-1.69)
*Women*	1.21 (0.84-1.76)	1.55 (0.92-2.59)	1.28 (0.82-1.98)
*Men*	0.86 (0.53-1.37)	1.10 (0.59-2.03)	1.08 (0.65-1.78)
Difference women/men^c^	*ns*	*ns*	*ns*
	**Model II**		
**Job Demands** *(ref. low)* ^a^	OR (CI 95%)^b^	OR (CI 95%)	OR (CI 95%)
*Total*	1.06 (0.78-1.44)	1.11 (0.74-1.66)	0.74 (0.54-1.03)
*Women*	1.45 (0.97-2.16)	1.59 (0.88-2.88)	0.88 (0.55-1.41)
*Men*	0.68 (0.43-1.09)	0.74 (0.42-1.330)	**0.60** (0.38-0.95)
Difference women/men^c^	*	*ns*	*ns*
**Job Control** *(linear * ^d^ *, high values = low control)*			
*Total*	0.98 (0.81-1.19)	**1.36** (1.08-1.71)	**1.23** (1.01-1.50)
*Women*	0.92 (0.73-1.17)	1.22 (0.88-1.68)	1.15 (0.88-1.50)
*Men*	1.10 (0.80-1.51)	**1.54** (1.11-2.13)	1.32 (0.98-1.77)
Difference women/men^c^	*ns*	*ns*	*ns*
**High job strain** ^e^ *(ref. all others)* ^f^			
*Total*	0.97 (0.72-1.30)	1.33 (0.88-1.99)	1.10 (0.79-1.53)
*Women*	1.12 (0.76-1.64)	1.51 (0.89-2.57)	1.23 (0.79-1.91)
*Men*	0.77 (0.47-1.26)	1.09 (0.57-2.09)	0.94 (0.56-1.57)
Difference women/men^c^	*ns*	*ns*	*ns*

Results of binary logistic regressions. All analyses were adjusted for follow-up year and baseline characteristics: age, sex, physical work environment, hours worked during previous year, mental health, mobility, and an index based on all diseases and symptoms that were used to create the outcome. Model II was adjusted for all these factors and for level of education. The analyses were conducted separately for women and men. Results in bold: p value <0.05. ^a^For women, the reference category was women with low job demands. For men, the reference category was men with low job demands. ^b^Abbreviations: *OR* odds ratio, *CI* confidence interval, *ns* nonsignificant. ^c^P value for how the association between work-related stress and health problems differs between men and women; e.g., the interaction between sex and job demands: *p < 0.05, *ns* = *p* ≥ 0.05. ^d^In linear representation, the ORs indicate the change in odds of each higher category of the independent variable. Job control has four categories. ^e^High job strain: control was dichotomized into low and medium low vs. high and medium high. High strain = high demands and low control. ^f^For women, the reference category was women who did not have high job strain; i.e., who had passive (low demands/low control), active (high demands/high control), or low strain (low demands/high control) jobs. For men, the reference category was men who did not have high job strain, i.e., who had passive, active, or low strain jobs.

After adjustment for level of education, the association between high job demands and lower odds of cognitive difficulties among men remained significant. Among men, low job control remained significantly associated with mobility limitations. When measuring women and men together, low job control remained significantly associated with both cognitive difficulties and mobility limitations in old age.

### Educational attainment and work-related stress

High job demands among men with low education were statistically significantly associated with lower odds of serious problems in one health domain and complex health problems (i.e., serious problems in at least two health domains). High job demands among women with low education were associated with higher odds of complex health problems (not statistically significant) (Table 4). However, the differences between women and men were significant (*p* < 0.001) (results not presented).

**Table 4:** **Interactions between work-related stress and educational attainment associated with domains with serious health problems**

	Number of domains with serious health problems ***(ref. no serious problems)***					
	1 domain			2-3 domains (Complex health problems) ^a^		
WORK-RELATED STRESS	Low education ^b^		High education	Low education		High education
	OR (CI 95%) ^c^	***p*** ^d^	OR (CI 95%)	OR (CI 95%)	***p***	OR (CI 95%)
**Job demands** *(ref. low demands)* ^e^						
Women	1.14 (0.69-1.88)	*ns*	0.95 (0.51-1.79)	1.65 (0.80-3.42)	*ns*	2.34 (0.71-7.70)
Men	**0.45** (0.29-0.78)	*ns*	0.81 (0.44-1.51)	**0.33** (0.14-0.81)	*ns*	1.18 (0.39-3.61)
**Job control** *(linear * ^f^ *, high values = low control)*						
Women	0.88 (0.62-1.24)	*ns*	1.07 (0.80-1.43)	1.16 (0.70-1.92)	*ns*	1.25 (0.82-1.88)
Men	**1.66** (1.06-2.58)	*	0.92 (0.67-1.26)	1.00 (0.55-1.80)	*	**2.19** (1.47-3.26)
**High job strain** ^g^ *(ref. all others)* ^h^						
Women	1.17 (0.71-1.92)	*ns*	1.18 (0.63-2.23)	1.71 (0.86-3.40)	*ns*	1.51 (0.62-3.66)
Men	0.69 (0.40-1.21)	*ns*	0.76 (0.38-1.51)	0.42 (0.17-1.02)	**	**3.07** (1.19-7.93)

Results of multinomial logistic regressions. All analyses were adjusted for follow-up year and at baseline: age, sex, physical work environment, hours worked during previous year, an index based on all diseases and symptoms that were used to create the outcome, mental health, and mobility. The analyses were conducted separately for women and men. Results in bold have p value <0.05. ^a^Those with serious problems in two or three health domains were classified as having complex health problems. ^b^Level of education was dichotomized as low education (compulsory; 6-8 years for most of the study participants) and high education (vocational, upper secondary, and university). ^c^Abbreviations: *OR* odds ratio, *CI* confidence interval, *ns* nonsignificant, *p* p value. ^d^P value for how the association between work-related stress and health problems differ by level of education: *p < 0.05, **p < 0.01, *ns* = *p* ≥ 0.05 ^e^For women, the reference category was women with low job demands. For men, the reference category was men with low job demands. ^f^In linear representation, the ORs indicate the change in odds of each higher category of the independent variable. Job control has four categories. ^g^High job strain: control was dichotomized into low and medium low vs. high and medium high. High strain = high demands and low control. ^h^For women, the reference category was women who did not have high job strain; i.e., who had passive (low demands/low control), active (high demands/high control), or low strain (low demands/high control) jobs. For men, the reference category was men who did not have high job strain, i.e., who had passive, active, or low strain jobs.

Low job control was significantly associated to higher odds of serious health problems in one domain among men with low education and to higher odds of complex health problems among men with high education. The interaction between job control and education in the association with serious problems in one health domain and complex health problems was statistically significant among men (Table 4). Furthermore, level of education correlated with job demands (r = .24, *p* < 0.001) and with job control (r = .54, *p* < 0.001) when adjusted for sex (not presented in a table).

High job strain was associated with complex health problems as shown in Table 2, and the interaction between high job strain and level of education was statistically significant in the association with complex health problems among men (Table 4). Among men with high education, high job strain was associated to increased odds of complex health problems. Among women, the general pattern was of an association between high job strain and serious health problems (in one or two to three domains) in old age regardless of level of education. The results, however, were not statistically significant (Table 4).

## Discussion

The main results of the study can be summarized as follows: job control was negatively associated with serious problems in one health domain and with complex health problems (having serious health problems in 2-3 health domains), with the strongest associations observed for impaired mobility and cognition. While not all results were statistically significant, high job demands were associated with higher odds of serious health problems among women but lower odds of serious health problems among men. The strongest association in this study was between high job strain and complex health problems.

After adjustment for level of education (a measure of socioeconomic position), some associations became statistically nonsignificant, suggesting that factors reflected in educational attainment may explain the link between greater work-related stress and greater likelihood of serious health problems in old age, whether in one single domain or in two or three domains (what we call complex health problems). High job demands remained related to lower odds of serious problems in one health domain among men, and low job control remained associated with higher odds of complex health problems among men. Having had high job demands was associated with lower likelihood of complex health problems among men with low education but not among men with high education or women regardless of level of education.

In this study, we had the rare opportunity to explore the potential effects of previous psychosocial working conditions using data with over twenty years of follow-up. Earlier studies, however, have found associations between work-related stress and health in old age that is in line with our results. For example, earlier research has shown that a low level of job control is associated with higher rates of multiple health problems in old age [26], that high job demands are linked with worse mental and physical health after retirement [25], and that low job control and high job strain are independently associated with cognitive decline [22–25].

Although many studies have looked at sex differences in associations between work-related stress and health where these variables were assessed at the same time, few studies have explored sex differences when health is assessed past working age. In this study, we found no statistically significant differences between the sexes in the association between job control and serious health problems in old age. However, we found that high job demands were associated with decreased odds of serious problems in one health domain in men. In women, high job demands were associated with an increase in the odds of complex health problems. This is in line with the previous findings suggesting that women may be more affected by psychological job demands than men [6]. Specifically, time pressure at work was associated with better health among men and worse health among women in terms of musculoskeletal pain [6]. In addition, as shown in Table 2, the protective effect found among men in regard of the association between job demands and serious health problems is notable in all the three health domains, although it is only significant for cognition. It may be that any effects of stress as a function of job demands in men are offset by intellectual engagement reflected in job demands. Alternatively, men may consider psychologically demanding work from a different perspective than women. This possibility may deserve further study. Moreover, the decreased odds of serious health problems among men with high job demands may partly be due to health selection.

Psychosocial workload has increased during recent decades. Work has become more mentally demanding and less physically demanding; hence, the experience of emotional and mental demands has increased [38, 39]. Since baseline data in this study ranged from 1968 to 1991, the results may underestimate work-related stress in today’s workforce and the potential repercussions of this stress for health in old age. A 1991 comparison between 12 European Union countries found that the self-reported level of control in the work environment was approximately the same in northern, middle, and southern Europe, but psychosocial job demands were slightly lower in northern Europe [40]. This suggests that our conclusions about associations between work-related stress in midlife and health in old age may be generalizable to other European Union countries and perhaps even to other developed countries, although they may underestimate the impact of job demands on serious health problems in old age.

One of the strengths of this study was the inclusion of a combination of a broad range of serious health problems in old age, whereas earlier research primarily focused on single health problems. The inclusion of a broader range of problems in this study enabled us to better capture the complexity of health problems in the older population. We made our definition of serious health problems rather restrictive and in order to exclude minor health problems we used high thresholds for serious problems within each health domain. Nearly 11 percent of the participants in our sample had complex health problems (i.e., serious problems in two or three health domains), i.e., they comprised a very vulnerable subset of the older population.

After adjustments for level of education (a measure of socioeconomic position), almost all associations became statistically nonsignificant. This may indicate that the association between work-related stress and health in old age is partly explained by educational attainment. However, this study shows that there are also independent associations between work-related stress and serious health problems in old age. Although educational attainment explains much of the association between work-related stress in midlife and health in old age, promoting preventive strategies to improve health at work is a more obtainable goal than changing educational attainment during adulthood. Therefore, the development of interventions that reduce work-related stress may still be the preferred target for intervention. Of note is that socioeconomic position may act as an “allocation mechanism”, hence reflecting individuals’ inherent risk for early onset of morbidity [41]. Given the role of educational attainment in the work stress-health links observed here, this inherent risk still interferes with the effectiveness of interventions concerning work-related stress.

Although not statistically significant, there was a general pattern of an association between high job demands, among men with low education and lower odds of serious health problems in old age, and higher odds among women regardless of level of education (Table 4). These results suggest that unmeasured heterogeneity between these groups may confound the associations. For example, it is well known that women and men tend to work in different types of occupations, and aspects of their occupations that were not measured in our study may be associated with both psychosocial working conditions and health in old age. Moreover, selective mortality impacts all studies of health among the oldest old: samples include only those healthy enough to survive into old age. Because those most severely affected by a risk factor may have died before the study started, the impact of the risk factors may appear to be smaller in magnitude than it actually is. This may lead to the appearance of decreased associations with earlier risk factors, as those whose health was most severely affected may already have died. The sex differences between work-related stress and health in old age could be affected by a stronger survival bias among men due to their overall higher mortality rates [42].

From a policy perspective, it is important to investigate potentially modifiable predictors of complex health problems in old age since these are strongly related to the amount of medical and social care needs in the population as well as the need for increased coordination and collaboration between different providers of medical care and social services [33, 43, 44]. From the perspective of our findings, paying special attention to the health in occupations characterized by high job strain, via policies targeting these workers with screenings and health promotion outreach may be particularly useful. Additionally, considering options to ease work stress on these workers may also be helpful.

Many governments are proposing higher retirement ages to help meet the economic challenges associated with the aging population. As poor working conditions in midlife increase the risk of labor market exit, investments in favorable working conditions have been proposed as one way to reduce rates of early retirement by preventing ill-health [45, 46]. In addition, an extended working life and/or working to older ages may strengthen the influence of midlife working conditions on health in old age. Therefore, the effects of work-related stress on health in old age are important from the economic, sociopolitical, and general health perspectives.

The study had some limitations. First, although non-response rates were low in the baseline studies and the follow-up waves, it is still possible that selection bias caused by non-response could affect the results. It is often not possible to interview the frailest old people. However, the use of proxy interviews partially compensated for selective non-response [47] and minimized exclusion of the oldest old [48]. A related issue is that the long gap between baseline and follow-up presents some advantages with respect to the assessment of long-term effects of work on health, it also has limitations, particularly as it offers only a snapshot of health in older age and does not allow for continuous tracking of health across the life course. Second, it is possible that individuals aged 46+ at baseline who were working may on average be healthier than those in the same age group who were not working, the so called “healthy worker effect” [49], and therefore were excluded from the study sample. Third, our assessment of work stress is limited. Newer versions of measuring this construct include additional items, which were not available in our data. Similarly, we used only one assessment of occupation. Although there was very little work mobility in this cohort and we assessed individuals at the prime of their working careers, there is still a possibility that the data were subject to non-differential misclassification bias. As our measure is crude, a variance in work stress is unaccounted for. If these unaccounted differences in work stress are also associated with health, our models are likely to underestimate the associations. However, the fact that we found meaningful results with this relatively crude assessment of work stress and occupation may indicate that these results are robust. Fourth, we cannot control for previous health and/or life situations before participating in the baseline survey. We did, however, control for health status (including mental state) at baseline in order to minimize the effect of a direct association between the psychosocial work environment and health. However, if there were an undetected reverse causation in regard of health status and work condition, the results in this study would most probably be an underestimation of the true effect. Finally, we were unable to account specifically for the work-life balance. Although controlling for mental health is helpful in this respect, a specific consideration of the work-life balance should be the focus of future studies.

## Conclusions

In conclusion, the results of this study support the notion that psychosocial working conditions play a role in shaping health throughout the life course. The results underscore the importance of careful monitoring and mapping of the long-term health consequences of work-related stress. Such monitoring may serve as a springboard for the development of preventive strategies to improve public health both before and after retirement. Some of the associations between work-related stress and health in old age diminished after controlling for education attainment, a measure of socioeconomic position, which indicates that economic and social position within society largely explain the association between psychosocial work environments and health over time. Still, work-related stress, which was important in some respects even when educational attainment was controlled, may still be the preferred target for intervention during adulthood than educational attainment.
